# Commentary: Acute and late toxicity in prostate cancer patients treated with moderately vs ultra-hypofractionated radiotherapy

**DOI:** 10.3389/fonc.2026.1909842

**Published:** 2026-07-20

**Authors:** Toufic Eid, Tarek Al-Bitar, Osama Mohamad

**Affiliations:** Department of Radiation Oncology, American University of Beirut, Beirut, Lebanon

**Keywords:** genitourinary toxicity (GU), moderately hypofractionated radiotherapy (MHRT), prostate cancer, real-world evidence, ultra-hypofractionated radiotherapy

Cifuentes-Quin et al. report a useful real-world comparison of toxicity after moderately hypofractionated radiotherapy (MHRT) and ultra-hypofractionated radiotherapy (UHRT) in 229 patients with localized prostate cancer treated in Colombia (1). The study is an extremely useful contribution, particularly because real-world data from Latin America and low- and middle-income countries (LMICs) in general remain limited and because shorter radiotherapy schedules may have important practical advantages in resource-constrained settings. As our institution is currently developing a prostate UHRT program, we found this report particularly relevant. In addition to its clinical outcomes, the study offers useful practical insights for centers implementing UHRT in real-world settings. The authors are to be commended for reporting their outcomes and for acknowledging several limitations of their analysis. However, we believe that a few issues are important to consider when interpreting the lower adverse events seen with UHRT.

First, the recently published PACE-C trial is directly relevant to this comparison and should be discussed (2). PACE-C is the only phase 3 randomized trial comparing the same two schedules used in the present study. Among 1,192 patients with intermediate- and high-risk prostate cancer, acute grade >=2 genitourinary toxicity by Radiation Therapy Oncology Group (RTOG) criteria was essentially the same between arms, at 28% with UHRT and 27% with MHRT (p = 0.89), whereas acute grade >=2 genitourinary toxicity by Common Terminology Criteria for Adverse Events (CTCAE) grading was borderline higher with UHRT, at 34% versus 28% (p = 0.05). Acute grade >=2 gastrointestinal toxicity by CTCAE grading was higher with UHRT, at 17% versus 10% (p = 0.0011), with no significant difference in grade >=2 gastrointestinal toxicity when reported by RTOG criteria (13% vs. 11%; p = 0.53) (2). These randomized data are difficult to reconcile with the much lower acute toxicity reported by Cifuentes-Quin et al. for UHRT compared with MHRT, 11.63% versus 25.17%, respectively (p = 0.013). This suggests that the observed benefit may reflect baseline differences between groups rather than the fractionation schedule alone.

Second, it is unclear why fractionation schedule was not included in the multivariable analysis of late toxicity in Table 4 of the original study (1). Thus, it is difficult to know whether UHRT remained independently associated with lower late toxicity after adjustment. Clarifying the rationale for excluding fractionation from this model would strengthen the interpretation of the late toxicity results.

Third, the statistical model should be interpreted cautiously as an exploratory adjustment model rather than as a validated clinical prediction tool. Although the authors reported the Hosmer-Lemeshow test to assess calibration, they did not report overall model performance, discrimination, or classification accuracy. In particular, no receiver operating characteristic curve or area under the curve was provided to assess discrimination, and no confusion matrix or derived metrics such as sensitivity, specificity, positive predictive value, or negative predictive value were presented.

Fourth, the imbalance in baseline urinary medication use is a major concern. Alpha-blocker use was reported in 18.88% of patients in the MHRT group and in none of the patients in the UHRT group (p < 0.001). Because medication use was the strongest predictor of both acute toxicity and late toxicity, statistical adjustment may not be enough to fully separate the effect of baseline urinary morbidity from the effect of the treatment schedule.

Fifth, the sample-size asymmetry between the groups should also be highlighted. The MHRT cohort included 143 patients, whereas the UHRT cohort included only 86 patients. This smaller contemporary cohort inherently limits the statistical power of multivariable logistic regression to detect subtle differences in uncommon events, especially high-grade toxicities. It also increases the instability of UHRT event rates, because a small number of additional events can disproportionately change percentages and adjusted estimates.

Sixth, the technical delivery details are particularly important. Cifuentes-Quin et al. reported that fiducial markers were not used for UHRT, and that treatment relied on daily image-guided radiotherapy with verification of bladder and rectal status, standardized bladder filling and rectal preparation, and PTV margins of 5 mm, reduced to 3 mm posteriorly (1). This is a valuable practical message for LMIC centers: with rigorous preparation, daily CBCT, appropriate margins, and strict organ-at-risk constraints, prostate UHRT may be deliverable without invasive fiducial placement or dynamic tracking technology. However, this technical interpretation competes with a second explanation: selection bias. The lower toxicity in the UHRT group may partly reflect selection of patients with better baseline urinary function, smaller glands or lower treatment volumes, less medication use, or other favorable clinical features. Because real-time intrafraction motion data, prostate volume, and detailed target-volume comparisons were not reported, the study cannot clearly distinguish between the safety of the technical protocol and the effect of patient selection.

Seventh, the lack of prostate CTV volume data further limits interpretation. Prostate size and target volume are known contributors to urinary toxicity after radiotherapy, and an imbalance in prostate volume or treated volume between groups could partly explain differences in genitourinary outcomes (4). This limitation is especially relevant when interpreting low toxicity after UHRT without fiducial-based tracking, because smaller prostate and CTV volumes could reduce both geometric uncertainty and dose to adjacent urinary structures.

Eighth, ADT use also differed between groups. ADT was more common in the MHRT cohort than in the UHRT cohort (97.20% vs. 90.70%; p = 0.024). The effect of ADT on urinary outcomes is not straightforward. It can reduce prostate volume and may improve obstruction in some patients, but it has also been associated with acute urinary retention (5), worsening storage symptoms in selected patients (6), increased urinary bother and incontinence in population-based studies (7), and higher grade >=3 late radiation toxicity with long-term compared with short-term ADT (8). At minimum, ADT represents another imbalance that could have affected urinary and sexual toxicity.

Ninth, the difference in follow-up is substantial and is probably one of the most important limitations for late toxicity. Median follow-up was 62.5 months in the MHRT group and only 12.1 months in the UHRT group (p < 0.001). The original article also did not report the median time to late side effects in either cohort, and particularly not in the UHRT cohort. Without these temporal data, it is impossible to know whether the UHRT cohort had exceeded the usual time window required for late toxicities to manifest. In PACE-B, genitourinary toxicity after UHRT showed a characteristic flare around 12 months, followed by improvement by 24 months (3). With a median follow-up of only 12.1 months, the UHRT group in the present study may not have been followed long enough to capture the full pattern of late GU toxicity after UHRT. The large difference in erectile dysfunction rates, 13.29% with MHRT versus 1.16% with UHRT (p < 0.001), should also be interpreted cautiously, since erectile dysfunction often develops over years rather than within the first months after treatment.

In summary, Cifuentes-Quin et al. provide valuable real-world evidence supporting the feasibility of UHRT for prostate cancer in a Latin American setting. Their experience is also technically encouraging because it suggests that, in carefully selected patients and with rigorous bladder and rectal preparation, daily image guidance and appropriate margins may permit safe UHRT implementation even without fiducials or real-time tracking. However, the reported toxicity advantage of UHRT should be interpreted with caution. Baseline urinary morbidity, target volume, ADT exposure, sample-size asymmetry, lack of full model-performance reporting, and markedly shorter follow-up all limit causal interpretation. Most importantly, we would be cautious about framing UHRT as a less toxic treatment than MHRT. The strongest randomized evidence from PACE-B and PACE-C supports UHRT as a much more convenient regimen with non-inferior cancer control and broadly comparable toxicity, not as a clearly less toxic approach (2, 3). Longer follow-up and prospective studies from centers in LMICs, ideally including patient-reported outcomes, standardized dosimetric reporting, and time-to-event toxicity analyses, will be important to confirm these findings and better define the role of UHRT in routine practice.

## References

[1] Cifuentes-Quin J Garcia-Montañez YT Hurtado-Ortiz A Cruz J Lagares LC Olarte-Licht LA . Acute and late toxicity in prostate cancer patients treated with moderately vs ultra-hypofractionated radiotherapy. Front Oncol. (2026) 16:1843032. doi: 10.3389/fonc.2026.1843032 42255231 PMC13236506

[2] Tree AC Hinder V Chan A Tolan S Ostler P van der Voet H . Intensity-modulated moderately hypofractionated radiotherapy versus stereotactic body radiotherapy for prostate cancer (PACE-C): early toxicity results from a randomised, open-label, phase 3, non-inferiority trial. Lancet Oncol. (2025) 26:936–47. doi: 10.1016/S1470-2045(25)00205-0 40517778 PMC7617972

[3] van As N Griffin C Tree A Patel J Ostler P van der Voet H . Phase 3 trial of stereotactic body radiotherapy in localized prostate cancer. N Engl J Med. (2024) 391:1413–25. doi: 10.1056/NEJMoa2403365 39413377 PMC7616714

[4] Martin JM Richardson M Siva S Cardoso M Handmer M Sidhom M . Mechanisms, mitigation, and management of urinary toxicity from prostate radiotherapy. Lancet Oncol. (2022) 23:e534–43. doi: 10.1016/S1470-2045(22)00544-7 36455582

[5] Yang TK Wu CC Chang CH Muo CH Huang CY Chung CJ . Subsequent risk of acute urinary retention and androgen deprivation therapy in patients with prostate cancer: a population-based retrospective cohort study. Med (Baltimore). (2020) 99:e18842. doi: 10.1097/MD.0000000000018842 32049786 PMC7035125

[6] Bang WJ Kim H Oh CY Jo JK Cho JS Shim M . Clinical significance of prostate volume and testosterone reduction on lower urinary tract symptoms in patients with prostate cancer undergoing androgen deprivation therapy. Sci Rep. (2022) 12:18535. doi: 10.1038/s41598-022-21963-1 36323749 PMC9630373

[7] Kopp RP Marshall LM Wang PY Bauer DC Barrett-Connor E Parsons JK . The burden of urinary incontinence and urinary bother among elderly prostate cancer survivors. Eur Urol. (2013) 64:672–9. doi: 10.1016/j.eururo.2013.03.041 23587870 PMC3938018

[8] Zumsteg ZS Zelefsky MJ . Short-term androgen deprivation therapy for patients with intermediate-risk prostate cancer undergoing dose-escalated radiotherapy: the standard of care? Lancet Oncol. (2012) 13:e259–69. doi: 10.1016/S1470-2045(12)70084-0 22652234

